# Influence of Hydrodynamics and Hematocrit on Ultrasound-Induced Blood Plasmapheresis

**DOI:** 10.3390/mi11080751

**Published:** 2020-07-31

**Authors:** Itziar González, Roque Rubén Andrés, Alberto Pinto, Pilar Carreras

**Affiliations:** Group of Ultrasonic Resonators RESULT, ITEFI, National Research Council of Spain CSIC 1, 28006 Madrid, Spain; roque.andres@csic.es (R.R.A.); alberto.delcorral@csic.es (A.P.); pilarcarreras@hotmail.com (P.C.)

**Keywords:** ultrasounds, microfluidics, blood plasmapheresis, hematocrit, hydrodynamics, cell enrichment, acoustophoresis, microtechnologies

## Abstract

Acoustophoretic blood plasma separation is based on cell enrichment processes driven by acoustic radiation forces. The combined influence of hematocrit and hydrodynamics has not yet been quantified in the literature for these processes acoustically induced on blood. In this paper, we present an experimental study of blood samples exposed to ultrasonic standing waves at different hematocrit percentages and hydrodynamic conditions, in order to enlighten their individual influence on the acoustic response of the samples. The experiments were performed in a glass capillary (700 µm-square cross section) actuated by a piezoelectric ceramic at a frequency of 1.153 MHz, hosting 2D orthogonal half-wavelength resonances transverse to the channel length, with a single-pressure-node along its central axis. Different hematocrit percentages *Hct* = 2.25%, 4.50%, 9.00%, and 22.50%, were tested at eight flow rate conditions of Q = 0:80 µL/min. Cells were collected along the central axis driven by the acoustic radiation force, releasing plasma progressively free of cells. The study shows an optimal performance in a flow rate interval between 20 and 80 µL/min for low hematocrit percentages *Hct* ≤ 9.0%, which required very short times close to 10 s to achieve cell-free plasma in percentages over 90%. This study opens new lines for low-cost personalized blood diagnosis.

## 1. Introduction

Blood is a complex biological fluid containing approximately 45% cellular components suspended in plasma (5 billion cells per milliliter of blood) and it represents an active indicator of various pathological disorders [1,2]. Many medical diagnostic procedures require a reliable separation of plasma from blood as it includes proteins, metabolites, circulating nucleic acids (CNAs), and other biomarker organisms. An efficient detection of these analytes requires plasma to be completely free of cells to prevent the pollution of contaminants interfering with the accuracy of analyses. Therefore, efficient separation of plasma (plasmapheresis) is the first step for an accurate blood analysis in most of the diagnostic studies [3].

Microfluidic devices are highly convenient for applications involving daily monitoring of patient blood as they require low sample volumes, fast delivery, and low cost. In particular, the low-Reynolds numbers inherent in most microfluidic systems lead to laminar flows and strong shear rates associated to the parabolic flow profile that can be exploited to drive strong non-Newtonian effects. Over the last decade, unprecedented advances have been reported in developing novel microfabrication techniques and microfluidic devices for blood separation [4,5,6,7,8,9,10,11,12,13,14,15,16,17,18]. Microfabrication techniques have facilitated the proliferation of in vitro studies on blood flows where the use of microfluidic models addressed questions pertaining to the role of microvascular morphology [19,20], blood viscosity [21,22], and hematocrit [23], as well as RBC deformation [24,25,26]. In these studies, blood is resuspended to a desired hematocrit (*Hct*) level in a buffer solution (ranging from non-physiological values of 10% and lower, to near-physiological values of 35%–50% [21,27] and higher). A major common challenge in blood plasma separation devices is the external stress acting on the cells, which causes hemolysis and therefore makes diagnosis process difficult.

Ultrasounds have been reported as a successful method to achieve a noninvasive process of blood plasma separation based on cell enrichment processes induced by acoustic waves [28,29,30,31,32,33,34]. This label-free technology is based on a steady radiation force acoustically induced by the ultrasounds. It allows the simultaneous manipulation of large groups of cells, driving and collecting them at certain positions of acoustic equilibrium and leading to blood plasma separation. Moreover, ultrasonic separators present several advantages as cell separation techniques such as high cell viability, simple design, ease of fabrication, continuous operation, and low-cost fabrication.

The efficiency of the acoustic technology to perform blood plasmapheresis depends on several inter-dependent parameters, such as cell size, cell densities, and fluid compressibility. Other fluid properties are indirectly involved in the acoustically induced hydrodynamic force action, such as the fluid viscosity, which becomes a key variable parameter specifically associated to blood as a non-Newtonian fluid. Blood viscosity is defined by the hematocrit (volume percentage of red blood cells in blood), cell aggregation and its hydrodynamics. In particular, shear rates in microfluidic flows generate nonlinear variations of the apparent blood viscosity, decreasing with a rise of the flow rate [19,21,35,36,37,38,39,40].

Additionally, an increase of hematocrit reduces the distance between the cells, favoring their aggregation. Cell aggregates (stacks-of-coins-like cell arrays named ‘rouleaux’) enlarge the flow disturbances increasing significantly the blood viscosity [41,42,43,44,45,46,47,48,49]. This phenomenon is particularly important in microcirculation, since it can dramatically influence blood flow in microvessels [50,51]. Therefore, the non-Newtonian dynamics of blood is highly influenced by the RBC concentration, their deformability, and ability to aggregate, which are defined by hydrodynamic flow conditions.

The dynamic response can vary when blood is exposed to external forces. In particular, dynamics of blood exposed to ultrasounds is not easily predictable due to the inter-dependence and crossed-involvement of such parameters. This current study aims to clarify and help this prediction due to the lack of experimental analyses in the literature. Various studies of the literature previous to our work describe experiments that demonstrate the ability of the ultrasounds to perform plasmapheresis in blood samples and analyze the advantages of these processes over other conventional methods [29,30,31,32,33,34]. In 2015, the authors of the current paper analyzed the influence of the hydrodynamic conditions of blood samples on the efficiency of the processes of plasma separation by ultrasounds at a fixed hematocrit percentage of *Hct* = 4.5%, with successful results [52]. However, those experiments were incomplete because they did not provide information about the influence of the hematocrit or cell concentration in the blood samples on the results of acoustically induced blood plasmapheresis.

Addressing it, this study analyzed the influence of hydrodynamics on blood plasmapheresis effects induced by ultrasounds at a fixed hematocrit percentage of *Hct* = 4.5% (with a 10% dilution of blood samples in a biocompatible buffer). In the study, optimal plasma separation results were found for some flow rates at this fixed *Hct* percent, rather than a predictable improvement in separation with increasing flow rate. These results indicated the indirect influence of other parameters involved in these acoustic processes not considered, such as the hematocrit (which defines the number of cells and their distances). This is a non-Newtonian fluid whose viscosity depends on the number of cells, their distances and capacity of aggregation, and the hydrodynamic conditions, and it could influence the acoustic response of the blood. Therefore, further experimental studies would be very valuable to clarify this complex crossed-influence of parameters in order to optimize the acoustic technology performance.

In this paper, we present an experimental study of blood cell bulk dynamics in microfluidic capillaries at different flow rate conditions and hematocrit levels. A simple acoustophoresis technique is investigated in which blood cells are separated from plasma by ultrasonic 2D standing wave forces at a frequency close to 1 MHz in a square cross section glass capillary. In particular, we aimed to find optimal hydro-acoustic conditions to extract cell free blood plasma with highest quality in the shortest times. This paper compiles the experimental results obtained.

## 2. Materials and Methods

### 2.1. Principle of Operation in Acoustophoretic Plasmapheresis: Theory

Particles or cells in suspension exposed to acoustic waves experience entrainment effects associated to a nonlinear interaction between the incident wave and that one scattered by each particle, generating an acoustic radiation force. A particle with volume *V_p_*, density ρ*_p_*, and adiabatic compressibility β*_p_* exposed to a standing wave and much smaller than the acoustic wavelength *λ*, experiments a radiation force that can be expressed as [53]

(1) $$F_{rad}=-\frac{\pi P_{0}^{2}V_{p}\beta_{0}}{2\lambda }\varphi \left(\rho ,\beta \right)\sin\left(2kx\right)$$

where $\varphi =\frac{5\rho_{p}-2\rho_{0}}{2\rho_{p}+\rho_{0}}-\frac{\beta_{p}}{\beta_{0}}$ is the acoustic contrast factor, *P*_0_ the amplitude of the incident wave pressure, *ρ*_0_ and *β*_0_ the density and compressibility coefficients of the fluid, and “*x*” the distance from the particle position to the nearest node of pressure in the standing wave. According to this equation, the particles collect in parallel bands perpendicular to the sound wave direction, separated by a half wavelength distance (*λ*/2). The sign of φ indicates the motion of the particles either toward the nodes (*φ* > 0) or to the antinodes (*φ* < 0). Red and white blood cells have positive acoustic contrast factors, so the radiation force drive them the toward pressure nodes, where collect to continue circulating. Acoustic plasmapheresis relies on these blood cell enrichment processes, leaving plasma free of cells flowing in areas close to the channel walls.

The velocity of a particle or cell moving towards a pressure node can be obtained from Equation (1) by equating the average ultrasonic force acting with Stoke’s drag force, $F_{D}=-6\pi \eta R_{p}u_{p}$. Neglecting inertial effects for micrometer-sized spherical particles, the particle trajectory from any position is derived as

(2) $$x\left(t\right)=\frac{1}{k}\arctan\left\{\tan\left(kx\left(0\right)\right)e^{\frac{4\varphi }{9}{\left(kR_{p}\right)}^{2}\frac{E_{ac}}{\eta }t}\right\}$$

with the acoustic energy density: $E_{\mathrm{ac}}=\frac{P_{0}^{2}}{4\rho_{0}\cdot c_{0}^{2}}$. The acoustic drift time *t_drift_* taken by a particle to move from any initial position *x*(0) to any final position *x(t)* toward the pressure node can be also calculated as

(3) $$t_{drift}=\frac{3\eta }{4\Phi \left(kR_{c}^{2}\right)E_{ac}}\ln\left[\frac{\tan\left[k\cdot x\left(t\right)\right]}{\tan\left[k\cdot x\left(0\right)\right]}\right]$$

Thus, blood cells located at a distance of 350 μm from pressure node should require few seconds to achieve this position. In a channel with flowing blood samples, the acoustic drift time $t_{drift}$ must be shorter than the travel time of the cells during their circulation along the channel, $t_{flow}$, (defined by the flow rate) to reach the pressure node before leaving the channel: $t_{drift}<t_{flow}$. Otherwise, the cells do not have enough time to reach the position of acoustic balance driven by the radiation force. This time relationship imposes some restrictions on the sample flow rates and a compromise between diverse parameters included in Equation (1). This implies that an adequate combination of acoustic and hydrodynamic conditions is the key for successful results in processes of acoustic blood plasmapheresis for every specific device design.

### 2.2. Acoustophoretic Device

**Figure 1:**
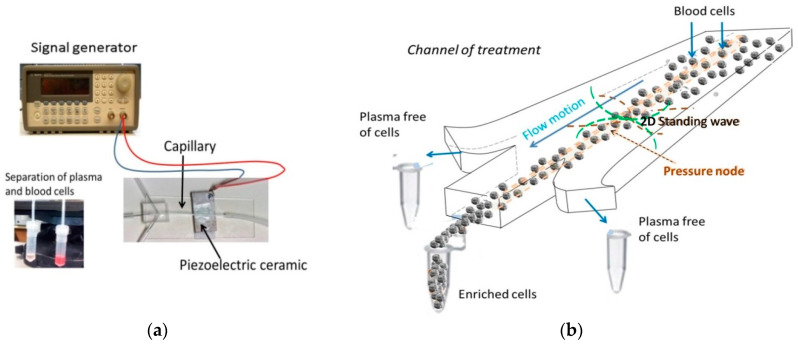
(**a**) Picture of the setup and (**b**) schematic of the plasmapheresis microchip actuated by ultrasound, governed by a resonance transverse to the channel length/flow motion.

A schematic of the proposed blood plasma separation device is shown in Figure 1. It consists of a borosilicate glass capillary with a squared hollow (CM Scientific Ltd., Silsden, West Yorkshire, UK) to which a piezoelectric actuator was attached underneath through a coupling hydrogel (Aquasonic Clear Ultrasound Gel, Parker Laboratories Inc., Fairfield, NJ, USA). A piezoceramic square ceramic pz26 (Ferroperm Piezoceramics, Kvistgard, Denmark) was used as acoustic transducer, with a thickness mode resonance at a frequency close to 1 MHz. It was actuated using a function generator (Agilent 33220A, Agilent Technologies Inc., Santa Clara, CA, USA) equipped with a power amplifier (E&I RF linear broad Amplifier 240 L, Research Blvd. Rochester, NY, USA).

Continuous sinusoidal waves were applied during the acoustic actuation with constant amplitude. The geometry and inner dimensions of the channel inside the capillary (0.7 × 0.7 × 50 mm^3^) allowed the establishment of a half-wavelength resonance within its cross section at a frequency *f* = 1.153 MHz, driven by the thickness mode resonance of the piezoelectric actuator. Two orthogonal standing waves were established in the cross section transverse to the channel length at this frequency, generating areas of maximal pressure beside the channel walls and pressure nodes in the central planes interfering along the central axis of the capillary.

The cells exposed to the ultrasounds were driven toward these nodes by the radiation force of Equation (1) along *x*- and *z*-directions, where collected, leaving the plasma around progressively free of cells.

The acoustically enriched cells in the center were extracted through a central outlet, separated from the plasma, which was released through the lateral outlets in a PDMS (polydymethylsiloxane) piece specifically manufactured for it [53], including the three outlets (230 × 230 µm^2^) attached to the end of the capillary.

Heparin treated blood samples obtained from healthy individuals (donor center of Hospital La Princesa, Madrid) were diluted in PBS (Sigma Aldrich, Spain) at rates 1:20, 1:10, 1:5, and 1:2 respectively, providing blood concentrations *C_Vblood_* = 5%, 10%, 20%, and 50% and hematocrit percentages of *Hct* = 2.25%, 4.5%, 9.0%, and 22.5% respectively (assuming a 45% volume of cells in whole human blood samples). The samples were infused by a syringe pump (Kd Scientific Syringe Pump, Holliston, MA, USA) into the capillary at different flow conditions varying from 20 µL/min up to 80 µL/min and exposed to the acoustic waves, and also quiescent blood samples were tested in rest. A fixed voltage of *V_P-P_* = 48 V was supplied to the piezoelectric actuator at *f* = 1.153 MHz to keep all the blood samples at the same acoustic conditions within the capillary in all the experiments, at any flow rate and blood hematocrit percentage. All the tests were performed applying the ultrasound on the blood samples for a duration of 22 s.

The small size of the capillary cross section and its dimensions intentionally coincident with *λ*/2, make impossible to measure the sound pressure inside the channel, even with the finest needle hydrophone of the market. Instead, we proceeded to a 3D numerical analysis to have an estimate of the spatial pressure distribution inside in the range of frequencies of interest. was numerically analyzed using the finite element method (FEM) software COMSOL Multiphysics^®^, version V-5.2.

## 3. Results and Discussion

**Figure 2:**
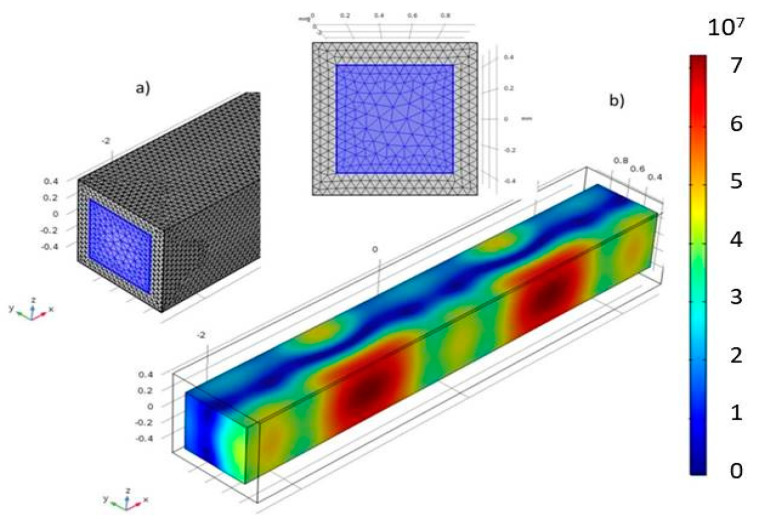
(**a**) 3D FEM modelling of the capillary; (**b**) COMSOL Multiphysics simulation of an acoustic pressure distribution inside the channel at a frequency *f* = 1.153 MHz Blue color refers to a null pressure amplitude.

In this analysis, the walls of the capillary were modeled with properties of Pyrex glass and thickness of 145 µm. The FEM simulation required the domains to be divided into a number of elements with size much smaller than the acoustic wavelength. Free tetrahedral cells were designed in the cavity (Figure 2a). A harmonic excitation in one of the walls of the capillary was assumed as the actuation of the ultrasonic transducer attached. In this simulation, properties of pz26 piezoelectric ceramics for the actuator (the same as that one used in the experiments), the blood properties as a fluid contained by the capillary were assumed.

In the analysis, a pressure nodal plane was found in the middle of the capillary cross-section. along its length at a frequency of *f* = 1.153 MHz (dark blue area in Figure 2b). This frequency was taken as reference in the experiments to find particle collection in this central plane of the capillary.

### 3.1. Experimental Results

Blood cells, exposed to a horizontal standing wave, deviated from their trajectories to the pressure plane established along the center of the channel where they collected, leaving a volume of plasma progressively free of cells (Supplementary Video S1) around. Thus, separation of plasma from blood cells was initiated by the combined effect of the acoustic forces and capillarity. The enrichment of the blood cells along the center of the capillary facilitated their extraction through a central outlet, while plasma was released through two lateral outlets symmetrically displayed in the PDMS piece attached at the end of the capillary described above (Figure 1).

Processes of cell collection along the central axis of the capillary were filmed and quantified at different blood dilutions and flow rate conditions. The present study aimed to quantify the dynamic response of blood in ultrasound-induced blood plasma purification processes by filming and processing images of the blood cells inside the capillary during the acoustic treatment. The blood plasmapheresis performance was analyzed by the progress in images transparency over time for the different blood dilutions, and acoustic and hydrodynamic conditions of the experiments. These processes were filmed at different blood dilutions and flow rate conditions. The increasing transparency of the images over time was analyzed for the different blood dilutions at the different acoustic and hydrodynamic conditions of the experiments.

**Figure 3:**
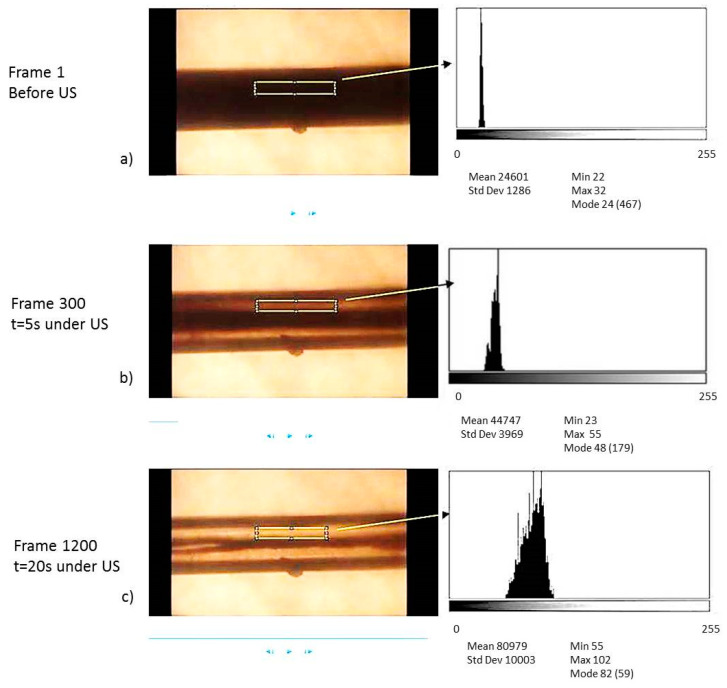
Three frames extracted from a movie of blood exposed to ultrasounds in the capillary (**a**) before the acoustic actuation; (**b**) at 5 s of acoustic exposure; (**c**) 20 s exposed to the ultrasounds at *f* = 1.153 MHz.

A rectangular area of observation of 0.16 mm^2^ was selected in the first frame of each movie, focusing part of the channel, parallel to the channel walls and excluding the central area where cells collect (approximately 200 µm-height and a 800 µm-width). This analysis area was kept fixed throughout all the film frames, showing a temporal a progression from dark to clearer images of the pixels of the focused area (Figure 3). The evolution of these properties during the acoustic actuation was quantified with the freeware Science image/NIH.

A 100% of transparency was assigned to samples containing only plasma free of cells (highest clarity of the focused area without cells).

### 3.2. Sampling and Quantified Estimation of Cell Release from Plasma in Areas of the Channel Out of the Center

**Figure 4:**
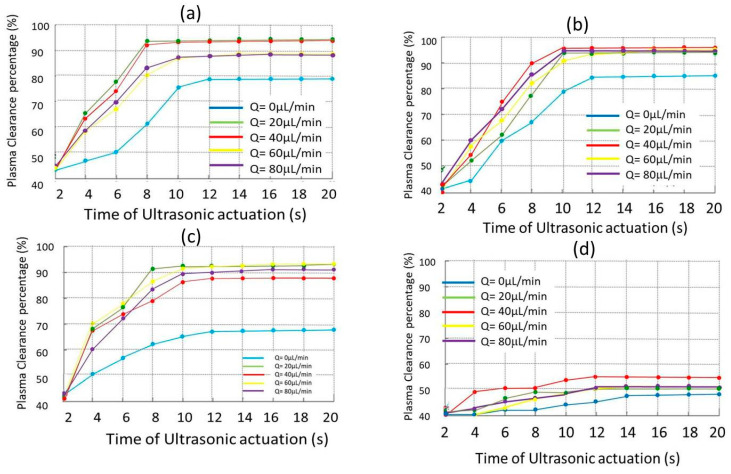
(**a**) Clearance blood plasma vs. time of acoustic treatment at different flow rate conditions for blood dilution samples: (**a**) 1:20; (**b**) 1:10; (**c**) 1:5 and (**d**) 1:2 respectively.

The percentage of plasma clear of cells progressively achieved during the acoustic actuation was calculated on each filmed image as $\left(1-C_{0}/C_{i}\right)\times 100\left(\%\right)$ where *C_i_* refers to the measured opacity level measured in the *i*-frame (time $t_{i}=i\times \frac{1}{capture\;velocty}$) and *C_0_* to the opacity level of the first frame. Although the opacity is a parameter not linearly related to the number of cells, it can be quantified taking as reference a value *C_ref_* = 0 for plasma fully free of cells. This parameter is proportional to the cell concentration in the filmed area and can be quantified, as done in our experiments. Higher cell concentration, darker images. Different evolutions on the transparency progression processes were found on the samples exposed to the ultrasounds until reaching a saturation that depended on the blood hematocrit and flow conditions. Figure 4a–d shows these quantified results as obtained from the filmed movies of the experiments.

Each graph of the four curves describes the clearance evolution over time for a determined hematocrit percentage at different flow rates ranging from quiescent blood samples *Q* = 0 µL/min up to *Q* = 80 µL/min while exposed to the acoustic field for 22 s.

The samples in rest (*Q* = 0 µL/min) exposed to the ultrasounds required longer times in their plasma clearance processes than blood in motion for any dilution tested (blue curves in the graphs). These stagnant samples showed a slow progression of plasma clearance to reach efficiency levels lower than 80%, always below those achieved by blood samples circulating along the capillary during the acoustic treatment. The cells at rest showed a high resistance to follow the acoustic drift motion towards the pressure node located in the central axis of the capillary. These findings are consistent with the non-Newtonian properties of blood, whose viscosity is defined by the aggregation capacity of cells, maximum in samples without circulation. In addition, cells adhered to the capillary walls were found in this hydrodynamic condition, which partially disturbed the images and their processing and, consequently, provided some uncertainty in the clearance results. This cell behavior was observed repeatedly in the quiescent blood samples exposed to the ultrasounds.

At high dilution levels (1:20, 1:10, and 1:5 corresponding to *Hct* = 2.25%, 4.5%, and 9.0% respectively) the samples required very short times of few seconds (*t* ~ 12 s) to culminate their blood plasmapheresis processes under the ultrasonic actuation at any flow rate below *Q* = 100 μL/min. These samples experienced significant changes in their acoustic response during the first 8–10 s of acoustic treatment to reach plasmapheresis percentages over 80% at any flow rate with the exception of quiescent samples, which despite the low hematocrit present high cell aggregability. They reached very high clearance percentages over 90%, even 96% for hematocrits of 4.5% and 2.25% at flow rates of 20 μL/min, 40 μL/min, 60 μL/min, and 80 μL/min respectively (green, red, yellow, and purple curves in Figure 4a–c). Optimal flow rates between 20 µL/min and 60 µL/min were found for these samples to perform the blood plasmapheresis in short times of acoustic actuation of 10–12 s. Our results revealed a low influence of the flow rate on the plasmapheresis processes at the low hematocrit percentages of *Hct* ≤ ~9% (assuming 45% for whole blood), reaching very high clearance percentages in very short times of less than 12 s. On the contrary, the acoustic treatment was useless to perform plasmapheresis on blood samples with *Hct* ~22.5% (dilution 1:2), revealing a strong inertial resistance of the cells to follow the acoustic drift motion at any flow rate, requiring times of at least three minutes to complete their plasmapheresis process, one order of magnitude higher than the others described above. Thus, the influence of the hematocrit on the acoustic response of the blood is more evident at the highest *Hct* percentage tested in the experiments (*Hct* = 22.5%) when comparing Figure 4d with the other three graphics of the figure. Our experiments have revealed a high influence of the cell concentration on the acoustic response of blood. Second order mechanisms associated with very short distances between cells *d_c-c_* produced at high hematocrit percentages (of the order of their diameters) which could be involved in the inability of the acoustic field to drive them toward the pressure node in the short periods of time of our experiments (22 s of acoustic treatment) which, in turn, is very effective on highly diluted samples with larger cell distances. Disturbances of the incident pressure around each cell by other nearby cell scattered waves [54,55,56] could acquire magnitudes close to those of the primary radiation force of Equation (1) at certain hematocrit levels.

In addition, other parameters related to the hematocrit seem to be indirectly involved in the cell response, such as the viscosity. This is a property related to the ability of aggregation of the blood cells, strongly influenced by their distance, in turn determined by their concentration (hematocrit) and mobility (flow rate). The cell concentration (*Hct*) determines the distance between them and, therefore, their ability to interact, which in turn produces changes in the blood viscosity. These factors determine the dynamic response of blood cells to acoustic waves.

After plasma cell acoustic separation, blood cells and plasma were extracted at the end of the capillary through different outlets. The purified plasma extracted through the lateral outlets was viable for later biomolecular analysis. The protein concentration was measured in a volume of 2 μL of the extracted plasma by a NanoDrop 2000c spectrophotometer (NanoDrop Technologies, Wilmington, DE, USA). PBS buffers used in each protocol were used as controls for optical density (OD) measurements. These results were comparable with another centrifuged blood plasma sample of the same dilutions and volume. Both results were also comparable to the theoretical composition of protein in human plasma (50–70 mg/mL) which in a dilution of 5% would correspond to a reading of 2.5–3.5 mg/mL.

## 4. Conclusions

This work analyzes the double influence of hematocrit (cell concentration) and hydrodynamics on the response of blood to external forces generated by ultrasounds in processes of acoustic blood plasmapheresis.

The experiments were performed in a glass capillary where a half-wavelength standing wave as established within its square cross-section at *f* = 1.153 MHz, providing a single node along its center. A resulting radiation force, parallel to shear forces induced by the 2D parabolic flow profile inside the capillary, drove the cells toward the center of the capillary.

Different dilution degrees of blood samples in PBS were used for the experiments, from 1:20 up to 1:2, with hematocrit percentages of *Hct* = 2.25%, 4.5%, 9.0%, and 22.5% respectively. The study was performed by analyzing the transparency in the filmed samples over time during the acoustic treatment at different hematocrit percentages and hydrodynamic conditions.

In the experiments, processes of blood plasma purification during the acoustic treatment were filmed and quantified. We aimed to find optimal correlations between acoustic and hydrodynamic conditions at the different hematocrit percentages.

The first conclusion of our results is that acoustic plasmapheresis should always be performed with samples circulating, not at rest, to prevent cell aggregation with an increase of viscosity. Any flow motion tested up to 80 µL/min demonstrated to be more favorable than quiescent conditions to perform acoustic plasmapheresis.

High dilutions up to 1:5 (*Hct* ≤ 9.0%) make processes of ultrasonic plasmapheresis very efficient in our microfluidic device. Percentages of blood plasma free of cells higher than 90% were achieved in very short times, between 10–12 s for samples flowing at rates up to 80 µL/min. However, higher flow rates and higher hematocrit levels show the ineffectiveness of the acoustic treatment in the acoustic conditions and time of acoustic actuation of our experiments of 22 s.

Shear forces established by microfluidic regime, radiation force, and acoustic streaming appear combined acting on the cells during the actuation of the ultrasounds.

Hydrodynamics are decisive to optimize acoustic plasmapheresis processes on highly diluted blood samples, as they provide the time of circulation of the cells within the channel, that must be always longer than the drift time required to reach the pressure node in the center of the capillary due to the acoustic radiation force, $t_{drift}<t_{flow}$.

A conventional 7.5-mL-volume of peripheral blood sample would require a time of 10.4 h to complete the acoustic plasmapheresis process in our device for 1:5 dilutions. However, it could be solved by replacing the capillary by a matrix of 10 capillaries connected in parallel directly attached to the same piezoelectric actuator, drastically reducing the treatment time to *t* = 1.04 h.

The extremely low-cost fabrication of our device opens a line of actuation for future clinical applications involving plasmapheresis in clinical trials.

Our results demonstrate that a selection suitable for the hydrodynamic conditions to optimize processes of blood plasmapheresis does not only depend on the acoustic conditions that define the radiation force acting on the cells, but they are also influenced by hematocrit. In particular, the viscosity increases with *Hct*, with the cell aggregation, and decreases with the flow velocity decrease, according to the non-Newtonian properties of blood.

Our experiments have revealed a cross-related influence on the results acoustic blood plasmapheresis, which could be related with the blood viscosity, defined by *Hct* and *Q*.

## Supplementary Materials

The following are available online at https://www.mdpi.com/2072-666X/11/8/751/s1, Video S1: the blood cell enrichment along the central axis of the capillary during the exposure of blood to ultrasounds.

## References

[1] Azim W., Azim S., Ahmed K., Shafi H., Rafi T., Luqman M. (2004). Diagnostic significance of serum protein electrophoresis. Biomedica.

[2] Blok S.L.J., Engels G.E., Van Oeveren W. (2016). In vitro hemocompatibility testing: The importance of fresh blood. Biointerphases.

[3] Kovarik M.L., Gach P.C., Ornoff D.M., Wang Y., Balowski J., Farrag L., Allbritton N.L., Gach P.C. (2011). Micro Total Analysis Systems for Cell Biology and Biochemical Assays. Anal. Chem..

[4] Shevkoplyas S.S., Yoshida T., Munn L., Bitensky M.W. (2005). Biomimetic Autoseparation of Leukocytes from Whole Blood in a Microfluidic Device. Anal. Chem..

[5] Haeberle S., Brenner T., Zengerle R., Ducrée J. (2006). Centrifugal extraction of plasma from whole blood on a rotating disk. Lab Chip.

[6] Yang S., Ündar A., Zahn J.D. (2006). A microfluidic device for continuous, real time blood plasma separation. Lab Chip.

[7] Mach A.J., Di Carlo D. (2010). Continuous scalable blood filtration device using inertial microfluidics. Biotechnol. Bioeng..

[8] Bhagat A.A.S., Bow H., Hou H., Tan S.J., Han J., Lim C.T. (2010). Microfluidics for cell separation. Med Boil. Eng..

[9] Nakashima Y., Hata S., Yasuda T. (2010). Blood plasma separation and extraction from a minute amount of blood using dielectrophoretic and capillary forces. Sensors Actuators B Chem..

[10] Dimov I.K., Basabe-Desmonts L., Garcia-Cordero J.L., Ross B.M., Ricco A., Lee L.P. (2011). Stand-alone self-powered integrated microfluidic blood analysis system (SIMBAS). Lab Chip.

[11] Hou H., Bhagat A.A.S., Lee W.C., Huang S., Han J., Lim C.T. (2011). Microfluidic Devices for Blood Fractionation. Micromachines.

[12] Bhardwaj P., Bagdi P., Sen A.K. (2011). Microfluidic device based on a micro-hydrocyclone for particle–liquid separation. Lab Chip.

[13] Zhang X.-B., Wu Z.-Q., Wang K., Zhu J., Xu J.-J., Xia X.-H., Chen H. (2012). Gravitational Sedimentation Induced Blood Delamination for Continuous Plasma Separation on a Microfluidics Chip. Anal. Chem..

[14] Nivedita N., Papautsky I. (2013). Continuous separation of blood cells in spiral microfluidic devices. Biomicrofluidics.

[15] Tripathi S., Prabhakar A., Kumar N., Singh S.G., Agrawal A. (2013). Blood plasma separation in elevated dimension T-shaped microchannel. Biomed. Microdevices.

[16] Nilghaz A., Shen W. (2015). Low-cost blood plasma separation method using salt functionalized paper. RSC Adv..

[17] Tripathi S., Kumar Y.V.B.V., Prabhakar A., Joshi S.S., Agrawal A. (2015). Passive blood plasma separation at the microscale: A review of design principles and microdevices. J. Micromech. Microeng..

[18] Xing X., He M., Qiu H.H., Yobas L. (2016). Continuous-Flow Electrokinetic-Assisted Plasmapheresis by Using Three-Dimensional Microelectrodes Featuring Sidewall Undercuts. Anal. Chem..

[19] Lima R., Wada S., Tanaka S., Takeda M., Ishikawa T., Tsubota K.-I., Imai Y., Yamaguchi T. (2007). In vitro blood flow in a rectangular PDMS microchannel: Experimental observations using a confocal micro-PIV system. Biomed. Microdevices.

[20] Kang Y.J., Ha Y.-R., Lee S.J. (2016). Deformability measurement of red blood cells using a microfluidic channel array and an air cavity in a driving syringe with high throughput and precise detection of subpopulations. Analyst.

[21] Kang Y.J., Yeom E., Lee S.J. (2013). A microfluidic device for simultaneous measurement of viscosity and flow rate of blood in a complex fluidic network. Biomicrofluidics.

[22] Yeom E., Kang Y.J., Lee S.-J. (2014). Changes in velocity profile according to blood viscos- ity in a microchannel. Biomicrofluidics.

[23] Shen Z., Coupier G., Kaoui B., Polack B., Harting J., Misbah C., Podgorski T. (2016). Inversion of hematocrit partition at microfluidic bifurcations. Microvasc. Res..

[24] Hou H., Bhagat A.A.S., Chong A.G.L., Mao P., Tan K.S.W., Han J., Lim C.T. (2010). Deformability based cell margination—A simple microfluidic design for malaria-infected erythrocyte separation. Lab Chip.

[25] Guo Q., Duffy S., Matthews K., Santoso A.T., Scott M.D., Ma H. (2014). Microfluidic anal-ysis of red blood cell deformability. J Biomech..

[26] Rodrigues R.O., Pinho D., Faustino V., Lima R. (2015). A simple microfluidic device for the deformability assessment of blood cells in a continuous flow. Biomed. Microdevices.

[27] Sosa J.M., Nielsen N.D., Vignes S.M., Chen T.G., Shevkoplyas S.S. (2014). The relationship between red blood cell deformability metrics and perfusion of an artificial microvascular network. Clin. Hemorheol. Microcirc..

[28] Hulström J., Manneberg O., Dopf K., Hertz H.M., Brismar H., Wiklund M. (2007). Proliferation and viability of adherent cells manipulated by standing-wave ultrasound in a microfluidic chip. Ultrasound Med. Biol..

[29] Lenshof A., Ahmad-Tajudin A., Järas K., Sward-Nilsson A.M., Aberg L., Marko-Varga G., Malm J., Lija H., Laurell T. (2009). Acoustic Whole Blood Plasmapheresis Chip for Prostate Specific Antigen Microarray Diagnostics. Anal. Chem..

[30] González I., Earl J., Pinto A., Fernandez L.J., Sainz B., Alcalá S., Monge R., Acosta V., Castillejo A., Soto J.L. (2018). A disposable thin chip for rapid isolation of tumor cells and aggregates by ultrasounds. Micromachines.

[31] Chen Y., Wu M., Ren L., Liu J., Whitley P.H., Wang L., Huang T.J. (2016). High-throughput acoustic separation of platelets from whole blood. Lab Chip.

[32] Harris N.R., Hill M., Beeby S., Shen Y., White N.M., Hawkes J.J., Coakley W.T. (2003). A silicon microfluidic ultrasonic separator. Sens. Actuators B Chemicial.

[33] Li P., Maoa Z., Peng Z., Zhoud L., Chena Y., Huanga P.H., Truicad C.I., Drabickd J.J., El-Deiryd W.S., Dao M. (2015). Acoustic separation of circulating tumor cells. Proc. Natl. Acad. Sci..

[34] Gu Y., Chen C., Wang Z., Huang P.-H., Fu H., Wang L., Wu M., Chen Y., Gao T., Gong J. (2019). Plastic-based acoustofluidic devices for high-throughput, biocompatible platelet separation. Lab Chip.

[35] Baskurt O.K., Meiselman H.J. (2003). Blood Rheology and Hemodynamics. Semin. Thromb. Hemost..

[36] Secomb T.W. (1987). Flow-dependent rheological properties of blood in capillaries. Microvasc. Res..

[37] Jaggi R.D., Sandoz R., Effenhauser C.S. (2007). Microfluidic depletion of red blood cells from whole blood in high-aspect-ratio microchannels. Microfluid. Nanofluid..

[38] Bodnár T., Sequeira A., Prosi M. (2011). On the shear-thinning and viscoelastic effects of blood flow under various flow rates. Appl. Math. Comput..

[39] Ikbal A. (2012). Viscoelastic blood flow through arterial stenosis—Effect of variable viscosity. Int. J. Non-linear Mech..

[40] Secomb T., Pries A.R. (2013). Blood viscosity in microvessels: Experiment and theory. Comptes Rendus Phys..

[41] Gester K., Jansen S.V., Stahl M., Steinseifer U. (2014). A Simple Method for the Investigation of Cell Separation Effects of Blood with Physiological Hematocrit Values. Artif. Organs.

[42] Reinke W., Gaehtgens P., Johnson P.C. (1987). Blood viscosity in small tubes: Effect of shear rate, aggregation, and sedimentation. Am. J. Physiol. Circ. Physiol..

[43] Meiselman H.J. (1993). Red blood cell role in RBC aggregation: 1963–1993 and beyond. Clin. Hemorheol. Microcirc..

[44] Pribush A., Meiselman H.J., Meyerstein D., Meyerstein N. (2000). Dielectric approach to investigation of erythrocyte aggregation. II. Kinetics of erythrocyte aggregation-disaggregation in quiescent and flowing blood. Biorheology.

[45] Bishop J.J., Popel A.S., Intaglietta M., Johnson P.C. (2001). Rheological effects of red blood cell aggregation in the venous network: A review of recent studies. Biorheology.

[46] Di Carlo D., Irimia D., Tompkins R.G., Toner M. (2007). Continuous inertial focusing, ordering and separation of particles in microchanels. Proc. Natl. Acad. Sci. USA.

[47] Meiselman H.J. (2009). Red blood cell aggregation: 45 years being curious. Biorheology.

[48] Wagner C., Steffen P., Svetina S. (2013). Aggregation of red blood cells: From rouleaux to clot formation. Comptes Rendus Phys..

[49] Baskurt O.K., Meiselman H.J. (2013). Erythrocyte aggregation: Basic aspects and clinical importance. Clin. Hemorheol. Microcirc..

[50] Mchedlishvili G., Varazashvili M., Gobejishvili L. (2002). Local RBC aggregation disturbing blood fluidity and causing stasis in microvessels. Clin. Hemorheol. Microcirc..

[51] Mchedlishvili G., Gobejishvili L., Beritashvili N. (1993). Effect of Intensified Red Blood Cell Aggregability on Arterial Pressure and Mesenteric Microcirculation. Microvasc. Res..

[52] González I., Carreras P., Ahumada O. (2015). Ultrasonic enrichment of flowing blood cells in capillars: Influence of the flow rate. Phys. Procedia.

[53] Gor’kov L. (1962). On the forces acting on a small particle in an acoustical field in an ideal fluid. Sov. Phys. Acoust..

[54] Raj M K., Chakraborty S. (2020). PDMS microfluidics: A mini review. J. Appl. Polym. Sci..

[55] González I., Gallego-Juárez J.A. (2003). Contribution of the acoustic-wake effect to the attenuation of sound in dilute suspensions of rigid particles. IEEE Trans. Ultrason. Ferroelectr. Freq. Control.

[56] González-Gómez I., Hoffmann T.L., Gallego-Juárez J.A. (2000). Theory and calculation of sound induced particle interactions of viscous origin. Acta Acust. United Acust..

